# Research priorities of the Canadian chiropractic profession: a consensus study using a modified Delphi technique

**DOI:** 10.1186/s12998-017-0169-4

**Published:** 2017-12-12

**Authors:** Simon D. French, Peter J. H. Beliveau, Paul Bruno, Steven R. Passmore, Jill A. Hayden, John Srbely, Greg N. Kawchuk

**Affiliations:** 1 School of Rehabilitation Therapy, Queen’s University, Louise D. Acton Building, 31 George St, Kingston, ON K7L 3N6 Canada; 2 Department of Chiropractic, Macquarie University, Sydney, NSW Australia; 3 Department of Public Health Sciences, Queen’s University, Kingston, ON Canada; 4 Faculty of Kinesiology and Health Studies, University of Regina, Regina, SK Canada; 5 Faculty of Kinesiology & Recreation Management, University of Manitoba, Winnipeg, MB Canada; 6 Department of Community Health & Epidemiology, Dalhousie University, Halifax, NS Canada; 7 Human Health & Nutritional Sciences, University of Guelph, Guelph, ON Canada; 8 Faculty of Rehabilitation Medicine, University of Alberta, Edmonton, AB Canada

**Keywords:** Chiropractic profession, Canada, Delphi study, Research priorities

## Abstract

**Background:**

Research funds are limited and a healthcare profession that supports research activity should establish research priority areas. The study objective was to identify research priority areas for the Canadian chiropractic profession, and for stakeholders in the chiropractic profession to rank these in order of importance.

**Methods:**

We conducted a modified Delphi consensus study between August 2015 and May 2017 to determine the views of Canadian chiropractic organisations (e.g. Canadian Chiropractic Association; provincial associations) and stakeholder groups (e.g. chiropractic educational institutions; researchers). Participants completed three online Delphi survey rounds. In Round 1, participants suggested research areas within four broad research themes: 1) Basic science; 2) Clinical; 3) Health services; and 4) Population health. In Round 2, researchers created sub-themes by categorising the areas suggested in Round 1, and participants judged the importance of the research sub-themes. We defined consensus as at least 70% of participants agreeing that a research area was “essential” or “very important”. In Round 3, results from Round 2 were presented to the participants to re-evaluate the importance of sub-themes. Finally, participants completed an online pairwise ranking activity to determine the rank order of the list of important research sub-themes.

**Results:**

Fifty-seven participants, of 85 people invited, completed Round 1 (response rate 67%). Fifty-six participants completed Round 2, 55 completed Round 3, and 53 completed the ranking activity. After three Delphi rounds and the pairwise ranking activity was completed, the ranked list of research sub-themes considered important were: 1) Integration of chiropractic care into multidisciplinary settings; 2) Costs and cost-effectiveness of chiropractic care; 3) Effect of chiropractic care on reducing medical services; 4) Effects of chiropractic care; 5) Safety/side effects of chiropractic care; 6) Chiropractic care for older adults; 7) Neurophysiological mechanisms and effects of spinal manipulative therapy; 8) General mechanisms and effects of spinal manipulative therapy.

**Conclusions:**

This project identified research priority areas for the Canadian chiropractic profession. The top three priority areas were all in the area of health services research: 1) Integration of chiropractic care into multidisciplinary settings; 2) Costs and cost-effectiveness of chiropractic care; 3) Effect of chiropractic care on reducing medical services.

**Electronic supplementary material:**

The online version of this article (10.1186/s12998-017-0169-4) contains supplementary material, which is available to authorized users.

## Background

Supporting research is an essential activity for contemporary healthcare professions. The Canadian chiropractic profession has demonstrated a commitment to support research through several initiatives, such as the provision of financial and other support to researchers with an interest in chiropractic [1]. As only limited funds are available to undertake research, existing funds may be better utilised if directed to areas of priority.

Research priorities can be established to address identified gaps in knowledge and to maximise opportunities to develop a relevant evidence base for a healthcare profession. Priorities may be identified at different strategic levels. These may be at the level of a research centre [2], at a national strategic level, such as a healthcare profession’s priorities [3, 4], or at an international level for specific clinical areas, for example low back pain [5], pediatric rheumatology [6] or musculoskeletal conditions in general [7].

In the United States and Europe, the chiropractic profession has developed research agendas that have been implemented with varying levels of success [8–15]. Previous efforts by the Canadian chiropractic profession to develop a research agenda occurred between the years 2000 and 2010 [1]. Initial research efforts were aligned with national Canadian health research priorities, and focused on three research streams: 1) Spinal biomechanics; 2) Neurophysiology; and 3) Epidemiology. Funding raised by the profession for research was directed toward supporting research-intensive appointments in Canadian universities for individuals with appropriate research training and a chiropractic background [1, 16]. More recently, the focus of the profession has shifted to the support of projects rather than people, so there is an immediate need for the Canadian chiropractic profession to develop a national research agenda.

The aim of this project was to identify research priority areas for the Canadian chiropractic profession, and to rank these in order of importance, based on the views of stakeholders in the chiropractic profession. The results of this study will be used by the Canadian Chiropractic Research Foundation (CCRF), the largest research funding body of research activity within the Canadian chiropractic profession, to inform funding decisions for future research.

## Methods

We conducted a modified Delphi consensus study [17, 18] to determine the views of representatives from Canadian chiropractic organisations and stakeholder groups about research priorities for the Canadian chiropractic profession. Three online Delphi survey rounds were conducted, and one face-to-face workshop, to generate and judge the importance of research areas. This was followed by a final online survey using a pairwise ranking activity to prioritise research areas in order of importance [19].

### Establishment of the Delphi panel

The Delphi panel was established with the aim to include representatives from all major stakeholder groups in the Canadian chiropractic profession. In consultation with Board members of the main stakeholder for this project, the CCRF, key organisations and groups were identified. Individuals from the following organisations and groups were invited to participate in the project, and included, where relevant, the Chief Executive Officer, the President/Chair, and Board Members:

1. CCRF
2. La Fondation Chiropratique du Québec
3. Canadian Chiropractic Association (CCA)
4. Canadian provincial chiropractic associations and regulatory authorities
5. Canadian Federation of Chiropractic Regulatory and Educational Accrediting Boards (Federation)
6. Canadian chiropractic educational institutions: Canadian Memorial Chiropractic College (CMCC); and Département de chiropratique, Université du Québec à Trois-Rivières (UQTR)
7. Specialty Canadian chiropractic colleges recognised by the Canadian Federation of Chiropractic Regulatory and Educational Accrediting Boards:
  - College of Chiropractic Sciences
  - College of Chiropractic Orthopedists (Canada)
  - Chiropractic College of Radiologists
  - Canadian Chiropractic Specialty College of Physical and Occupational Rehabilitation
  - Royal College of Chiropractic Sports Sciences (Canada)
8. Canadian Chiropractic Protective Association (CCPA)
9. Active researchers with a chiropractic background, including past and present chiropractic Research Chairs, and PhD students
10. Chiropractic student associations at CMCC and UQTR

### Delphi panel member recruitment

Eighty-five individuals representing these chiropractic organisations and groups were invited to participate in the Delphi panel. Invitations were sent by email in August 2015, with up to two reminder emails spaced 2 weeks apart. Administrative staff at the CCA office sent most invitation emails to potential participants on behalf of the research team. The CCA has a contact database that comprises email addresses of each of the people we approached for participation. If this was not the case, e.g. representatives of the CMCC and UQTR, the lead author sent these invitation emails directly. The invitation emails contained information about participating in the study, outlined the consent procedure for participation (consent implied by completion of the survey), and a link to the online survey. We also raised awareness of the study by making a presentation at a meeting where key chiropractic profession stakeholder groups were present.

### Delphi study procedure

The Delphi study consisted of three online surveys (created via the online platform FluidSurveys™, http://fluidsurveys.com/), and a face-to-face workshop. For each Delphi survey round, email invitations were followed up by two email reminders to non-responders.

In Round 1, participants were asked to suggest research areas that they considered important, and to provide a rationale for their suggestions, within each of the following broad research themes: 1) *Basic science*: studies investigating theories or mechanisms; 2) *Clinical research*: studies investigating patient outcomes; 3) *Health systems and services research*: Studies investigating access to health care, or the quality and cost of health care; 4) *Social, cultural, environmental and population health research*: studies investigating the health of the general population; and, 5) Other research areas.

Participants were asked to suggest research areas that they judged to be important, and important to the members of the organisation that they represented. If relevant, participants were asked to consult with the constituent members who they represented in order to determine which research priorities were important to them, e.g. practicing chiropractors who were members of their provincial association.

In each Delphi round, we asked participants to consider their suggestions based on priority criteria, adapted from a study undertaken in the United Kingdom physiotherapy profession [3]:

1. Does the topic address a significant need or gap in the evidence for Canadian chiropractic practice and/or service delivery?
2. What is the potential impact of the research for the quality of chiropractic care and the experience for patients, their caregivers, and members of the public?
3. What is the potential impact of the research for Canadian chiropractic practice?
4. What is the potential impact of the research for: Canadian chiropractic profession decision-makers; third party payors including insurance companies, workers and motor vehicle accident compensation authorities; and, relevance to government policy and priorities?

Results from Round 1 of the survey were presented to some participants in a face-to-face facilitated workshop at the CCA National Convention in September 2015. All survey participants were invited to attend the workshop, and 28 individuals attended. Participants were presented the results of Round 1 of the Delphi survey. Small groups, containing representatives from each of the stakeholder groups listed above, discussed the Round 1 results facilitated by a member of the research team. The small group task included a discussion of whether the initial research areas identified were comprehensive, and whether they met the research priority criteria. Members of the small groups could also identify new research priority areas not already suggested. Facilitators ensured that all small group members were given an opportunity to contribute to the discussion. Following the discussion, the facilitator collated any new research ideas, and these were added to the list of ideas identified in Round 1 of the Delphi survey.

All research priority areas suggested by participants were then collated by theme and further categorised into sub-themes. Sub-themes from the Round 1 responses, and from the workshop, were generated independently by two authors (PJHB and PB), and then discussed with the lead author to achieve consensus.

In Round 2 of the Delphi survey, we presented all of the sub-themes and research ideas to the participants, along with stated rationales from Round 1. Participants were asked the question “How important is the following research area for the chiropractic profession in Canada?” and were asked to rate the importance of the sub-themes on a Likert scale (essential; very important; moderately important; slightly important; not at all important). Participants could also suggest new research ideas and sub-themes during Round 2, and these additional areas would be rated for importance in Round 3. Using the results of Round 2, we defined consensus as 70% of the participants agreeing that a research priority was “essential” or “very important”. Although there is no universally agreed proportion for consensus for Delphi studies, 70% consensus is a commonly used benchmark [18].

In Round 3, the results of Round 2 were provided to the participants showing each research sub-theme and the percentage results for sub-themes that reached, and did not reach, consensus. We asked participants to re-rate the importance of each research sub-theme, taking into consideration the panel members’ importance rating as a whole.

### Prioritisation process

In the final survey round, all research priority areas that reached consensus in Round 3 were ranked by the participants via 1000Minds (1000Minds software, https://www.1000minds.com/) using conjoint analysis methodology [19]. Panel members were asked to rank pairwise statements by determining which of two randomly selected research sub-themes they considered more important for the Canadian chiropractic profession. The software eliminates other possible pairwise comparisons that have been implicitly ranked as corollaries of questions already answered, thus minimising the number of pairwise-ranking questions for each panel member. This was achieved by the ‘transitivity’ principle; for example, if a participant ranked sub-theme ‘A’ ahead of research area ‘B’ and also ‘B’ ahead of sub-theme ‘C’, then ‘A’ would be ranked ahead of ‘C’ and the software did not ask a question pertaining to this third pairwise ranking. Based on participants’ responses, 1000Minds uses mathematical methods (explained in detail in [19]) to arrive at overall rankings of the research sub-themes, both for each individual participant and also averaged across all participants. We present median and mean rank across all participants for each research sub-theme are presented.

## Results

Of the 85 individuals invited to participate, 57 (67%) accepted to form the Delphi panel and completed Round 1. Of those panel members who completed Round 1, 56 individuals (98%) and 55 individuals (96%) completed Rounds 2 and 3, respectively. Fifty-three panel members (93%) completed the pairwise ranking activity.

Demographic characteristics of the panel members are shown in Table 1. There was at least one panel member from each Canadian province, but no panel members from the Northwest Territories, Nunavut and Yukon. For those panel members who held a chiropractic degree (*n* = 52), the majority (69%) graduated from CMCC. Approximately half of the panel members indicated that they worked in clinical practice, half indicated that they worked in an educational institution (teaching and/or research), and one quarter indicated they worked in chiropractic administration. Panel members represented the broad range of chiropractic organisations that we approached for participation and, to maintain confidentiality, we did not ask participants to identify the particular organisation that they represented.

**Table 1:** Delphi panel participant demographic characteristics (*n* = 57), reported as n (%) unless otherwise stated^a^

Characteristic	
Age (mean, SD)	47. 5, 11.0
Female (1 response missing)	14 (25%)
Canadian province where reside	
Ontario	23 (40%)
Quebec	13 (23%)
Alberta	4 (7%)
British Columbia	4 (7%)
Manitoba	4 (7%)
Saskatchewan	3 (5%)
Newfoundland and Labrador	2 (4%)
Prince Edward Island	2 (4%)
New Brunswick	1 (2%)
Nova Scotia	1 (2%)
Northwest Territories, Nunavut, Yukon	0
Highest academic degree (1 response missing)	
Bachelor	7 (13%)
Doctor of Chiropractic	16 (29%)
Masters	7 (13%)
PhD	23 (41%)
PhD Candidate	3 (5%)
Holds a chiropractic degree	52 (91%)
Institution where received chiropractic degree	
Canadian Memorial Chiropractic College	36 (69%)
Université du Québec à Trois-Rivières	4 (8%)
Palmer College of Chiropractic	3 (6%)
Other^b^	9 (17%)
Years since graduating from chiropractic college (mean, sd)	23.3, 9.8
Place of work^c^	
Clinical practice	27 (47%)
Educational institution (teaching and/or research)	30 (53%)
Administration (including CEO/Executive Director)	14 (25%)
Other	8 (14%)
Organisation that participant represents^c^	
University	19 (34%)
Chiropractic association	17 (30%)
Chiropractic educational institution	16 (29%)
Chiropractic speciality college	8 (14%)
Regulatory board	7 (13%)
Funding body	3 (5%)
Student association	3 (5%)
Other	8 (15%)

^a^Due to rounding, may not add to 100%

^b^Cleveland Chiropractic College; Institut Franco-Européen de Chiropraxie; Logan College of Chiropractic; National University of Health Sciences; Northwestern Chiropractic College; New York Chiropractic College; University of Western States

^c^Participants could choose more than one response

In Round 1, including the online survey and the face-to-face workshop, 290 unique research ideas were generated by panel members. The research ideas were categorised into 31 sub-themes by the research team. The Additional file 1 shows all research ideas and stated rationales for these ideas, categorised by sub-theme. In Round 2, eight of these sub-themes were judged to be important by panel members. No new sub-themes were generated in Round 2. In Round 3, these same eight sub-themes were confirmed as important by the panel, and no sub-themes were changed from not important to important.

Table 2 summarises the results of the final Delphi round, and the priority ranking for each research sub-theme considered important. The top three ranked research sub-themes all fell under the theme of health systems and services research. No population health research sub-themes were considered important by the panel. Table 3 shows the sub-themes for which consensus on importance was not reached by the Delphi panel, suggesting these sub-themes are less of a priority.

**Table 2:** Final ranked list of priority research sub-themes, showing the percentage of the panel who rated each sub-theme as important in Delphi Round 3, and also showing the median and mean rankings in the final priority setting activity

Rank	Research sub-theme	Percentage^1^	Group Median Rank (Q1-Q3)^2^	Group Mean Rank (SD)^2^
1	*Health systems research:* Integration of chiropractic care into multidisciplinary settings	92%	3 (1.5–5)	3.55 (2.26)
2	*Health systems research:* Costs and cost-effectiveness of chiropractic care	94%	3 (2–5)	3.57 (1.93)
3	*Health systems research:* Effect of chiropractic care on reducing medical services	89%	4 (2–5.5)	4.08 (2.06)
4	*Clinical research:* Effects of chiropractic care	98%	4 (3–6)	4.33 (1.87)
5	*Clinical research:* Safety and side effects of chiropractic care	87%	5 (3.5–7)	4.98 (2.07)
6	*Basic science:* General mechanisms and effects of spinal manipulative therapy	85%	5.5 (2.5–7)	4.93 (2.45)
7	*Basic science:* Neurophysiological mechanisms and effects of spinal manipulative therapy	80%	5.5 (3–7)	5.12 (2.38)
8	*Clinical research:* Chiropractic care and older adults	80%	5.5 (4–7)	5.43 (1.87)

1. Percentage of panel rating sub-theme as important in Delphi Round 3

2. Obtained from the 1000Minds ranking activity of the research sub-themes, the median and mean represents the group median/mean ranking, with 1st (Q1) and 3rd (Q3) quartiles, and standard deviation (SD)

**Table 3:** Research sub-themes not considered important by the Delphi panel, grouped by research themes

Research sub-theme	Percentage of panel rating sub-theme as important in Delphi Round 3
Basic Science	
Biomechanical mechanisms and effects of spinal manipulative therapy	57%
Physiology of spinal pain	44%
Mechanisms of musculoskeletal disorders	33%
Mechanisms and effects of adjunct therapies	13%
Clinical research	
Identification of clinical sub-groups	63%
Patient satisfaction and expectations	63%
Dose-response of spinal manipulative therapy	57%
Maintenance/prevention care	57%
Development of outcome measures	49%
Effects of chiropractic care for non-musculoskeletal conditions	33%
Diagnostic testing of spinal conditions	33%
Chiropractic care for mental health	9%
Health systems research	
Access and barriers to chiropractic care	58%
Practice patterns, practice behaviours, and guideline implementation	55%
Third-party payers	46%
Chiropractic care versus other care	40%
Models of chiropractic care	34%
Chiropractic education/training	26%
Population health research	
Patient priorities	57%
Musculoskeletal population health	54%
Chiropractic care for special populations	33%
Management of comorbidities	28%
Determinants of health	19%

## Discussion

Eight research sub-themes were identified and prioritised using a multi-stage consensus process involving representatives of the key organisations in the Canadian chiropractic profession. These research sub-themes may be used as the basis for developing a research agenda for the Canadian chiropractic profession and to guide future coordinated research activities to maximise focus and impact.

The top three priority sub-themes were related to health systems and services research, reflecting that the panel felt the need to better understand the potential value and role that chiropractic services may play within healthcare. Two sub-themes in the clinical research area (Identification of clinical sub-groups; Patient satisfaction and expectations) were just below the threshold for consensus (63%). These sub-themes, despite not reaching the arbitrary cut-off of 70% consensus, could still be considered when formulating a national research agenda.

A high percentage of the Delphi panel identifying a research sub-theme as important did not necessarily mean that the theme was ranked highly when the priority process took place. For example, the sub-theme “Effects of chiropractic care” was identified as the most important by 98% of the panel members, but this sub-theme was ranked as only fourth most important sub-theme when participants chose between different sub-themes. Just because a sub-theme was rated as important, did not mean that the sub-theme was ranked as the *most* important.

A recent Delphi study within the chiropractic profession in Europe determined research priorities, and the following three items were thought to be most important: 1) Cost-effectiveness/economic evaluations; 2) Identification of subgroups likely to respond to treatment; and 3) Initiation and promotion of collaborative research activities [14]. Of these three priorities, only the first priority is similar to our results, possibly reflecting different cultural and political values between Europe and Canada, but may also be because the European study only included active researchers in their Delphi panel, whereas in our study we involved all major stakeholders of the Canadian chiropractic profession, including active researchers and political organisations.

Previous investigators have questioned the value of identifying research priorities for healthcare professions because there is little evidence that these processes have a direct effect on subsequent research output [20]. In view of the increasing challenges for healthcare researchers to obtain research funding, it is necessary that any prioritisation process is undertaken systematically, engages key stakeholders who can influence where future funding is directed, and identifies clear priorities that can be targeted with restricted research resources [21]. In our study, we achieved these aims through a comprehensive engagement process with the main research funding body of the Canadian chiropractic profession (CCRF), and involved participants from all major stakeholders. Our preliminary results were presented to these stakeholders in a community meeting, with the aim that the stakeholders will subsequently use these results to inform a national research agenda. The high-quality of the method we used gives the best possible chance for implementation.

The next step is for the Canadian chiropractic profession decision-makers to consider these priorities as they determine a national research agenda for supporting research activity. However, the identified priorities will need to be placed within a framework of available resources, timelines, and probability of success [22].

### Strengths and limitations

We used a robust consensus method to develop our research priorities, and invited representatives of the main stakeholders of the chiropractic profession in Canada to ensure wide representation. However, our overall response rate was low at 67%, leading to uncertainty about the representativeness of our sample. We did, however, achieve a high retention rate with 93% of original panel members completing the prioritisation activity.

The top two ranked sub-themes had a median ranking of 3, indicating that there did not appear to be a strong consensus for which sub-theme should be ranked highest. This is also highlighted with the percentage agreement of rated importance by the group not necessarily being consistent with the group ranking of individual research sub-themes. The order of the final ranked list of priority research sub-themes should be interpreted with caution.

We chose to not sample a random cohort of practising chiropractors in the Delphi study; we determined that the challenges of achieving a representative group of chiropractors in Canada was too difficult with our available resources. In order to gain as broad an input from the chiropractic profession as possible, participants were asked to canvass the views of their members, including practicing chiropractors; however, this was only a suggestion to participants and we did not measure if participants actually did this. Due to our recruitment process being conducted mainly by administrative staff at the CCA we do not have demographic information about non-responders, hence cannot determine if non-responders were different to responders. We did not implement any measures to include patients of chiropractors in the Delphi panel, so this is another limitation of our results; the research priorities of patients are unknown. Another limitation was the absence of patient groups as stakeholders. In an era where patient-centred care is a priority, including the voice of those who receive chiropractic care may have influenced the results of this study. Finally, we did not include people from outside of the chiropractic profession, e.g. research funders, health systems experts, and researchers from other disciplines.

## Conclusions

A national consensus process involving key stakeholders from the Canadian chiropractic profession has identified, and prioritised, eight research areas. The top three priority areas were all in the area of health systems research: 1) Integration of chiropractic care into multidisciplinary settings; 2) Costs and cost-effectiveness of chiropractic care; 3) Effect of chiropractic care on reducing medical services. The remaining five priority areas were in the area of clinical research and basic science research.

## Additional file

Additional file 1: All suggested research ideas and rationales categorised by sub-theme. (PDF 229 kb)
