# Supplementary material for: Research priorities of the Canadian chiropractic profession: a consensus study using a modified Delphi technique
Source: Chiropr Man Therap. 2017 Dec 12;25:38. doi: 10.1186/s12998-017-0169-4 (PMC5727882; doi:10.1186/s12998-017-0169-4)
Supplement: Additional file 1: — All suggested research ideas and rationales categorised by sub-theme. (PDF 229 kb) [file 12998_2017_169_MOESM1_ESM.pdf]

Appendix: Themes and sub-themes

Basic Sciences Research

General mechanisms and effects of SMT
Neurophysiological mechanisms and effects of SMT
Biomechanical mechanisms and effects of SMT
Mechanisms and effects of adjunct therapies
Mechanisms of musculoskeletal disorders
Physiology of spinal pain

Clinical Research

Effects of chiropractic care (general and for specific MSK conditions)
Effects of chiropractic care (non-MSK conditions)
Chiropractic care and older adults
Dose-response of SMT
Safety and side effects of SMT
Diagnostic testing of spinal conditions
Development of objective outcome measures
Patient satisfaction and expectations
Maintenance/prevention care
Identification of clinical sub-groups
Chiropractic care for mental health

Health Systems Research

Integration of chiropractic care into multidisciplinary settings
Costs and cost-effectiveness of chiropractic care
Effect of chiropractic care on reducing medical services
Chiropractic care vs. other care
Access and barriers to chiropractic care
Practice patterns, practice behaviours, and guideline implementation
Third-party payers
Chiropractic education/training
Models of chiropractic care

Population Health Research

Musculoskeletal population health
Management of co-morbidities
Determinants of health
Chiropractic care for special populations
Patient priorities

THEME: Basic Science Research

Sub-theme: General mechanisms and effects of SMT

Research Idea	Reason
Mechanism of action and effects of manipulation	*understanding how manipulation affects pain will assist in patient management and designing of clinical trials; further establish overall cause and effect on neuroimmune system, sensormotor changes; explain what chiropractors do and how SMT works to patients and stakeholders*
Intervention mechanism	*Understanding dose/response and intervention mechanisms allows clinicians to enhance clinical decision making process. Understanding what the treatment does or doesn't do is a critical step in deciding what treatment to provide to a patient.*
What mechanisms explain commonly reported Non-MSK outcomes?	*This is the area of practice where there are the most unanswered questions. Accoring to OCA member surveys the majority of practitioner, even those who consider themselves evidence based, believe there are impacts on internal organ funcation. This is also the primary criticism of the profession.*
What is the mechanism of impact of chiropractic care on non-msk conditions	
Further exploration of mechanisms using newer methodologies, approaches: Are we missing molecular, genetic, genomics, histopathology of healing (healing time), responders/non-responders, effect of multimodal care (not just SMT, combination care etc), injection therapies mechanisms	
Cellular response to shear, stretch and compression forces	*Variability in response to manipulation is likely mediated by variation in signalling pathways. Understanding which responses are likely and when provides new data to clinical scientists looking at a variety of patient outcomes.*
Under what mechanisms do different treatments work for disc pain?	*Several treatments for disc pain do not necessarily have a neurophysiological or biomechanical basis*
Controlled human experiments to determine whether spinal manipulative therapy (SMT) is associated with prolonged changes in biochemical markers (e.g. down-regulation of inflammatory mediators/cytokines)	*Need to determine if SMT has any clinically significant specific effects on inflammatory or otherwise painful MSK conditions (e.g. arthritis, acute sprains/strains)*
Cumulative effect of SMT on pain, spine stiffness, muscle activity, muscle force, balance, co-ordination etc.	*provide evidence for treatment plans (frequency)*
Mechanical stimuli affecting pro-inflammatory and anti-inflammatory cytokines.	*Again - there is a dearth of information to guide clinical sciences regarding the body's reactions to mechanical changes. Clinical sciences need this information in order to conduct new research and appropriately stratify their samples.*
How do various tissue/cell types respond to the kinds of forces generated during SMT.	*This is one specific direction for research into the neurobiological basis of SMT and is worthy of immediate attention because it is so practical to conduct this type of work immediately. So many techniques are based on formulaic approaches which can be dissected and tested.*
Tissue responses to different kinds of manipulation.	*Manipulation is a complex science for which there are an abundance of techniques - each of which may have a different response in different individuals. This variability has severely challenged clinical trials in manipulation. There is a great need for studies understanding the body's response to different forms of manipulation applied to different locations in the back (even within the same region), so that greater specificity can guide future trials work.*
Difference in mechanisms of different types of chiropractic adjustments (Gonstead, Diversified, Activator, Thompson, etc)	
Mechanism and theory behind the leg length analysis for spinal adjustments	
Develop a model of spontaneous stroke	*It appears that the neck manipulation/stroke situation is best described by the recognition that patients are undergoing what has been phrased as "spontaneous stroke". Mouse models can be developed to create mice who are likely to have a spontanous stroke. After the development of such mouse models, testing can be done in humans. If positive, a test can be developed for the population in order to identify those individuals who should not, for example, play football, and who may be at risk with a chiropractic manipulation of the neck. Such a study would show the intention of the chiropractic profession to lead in this very problematic area.*

Sub-theme: Neurophysiological mechanisms and effects of SMT

Neuro physiology of subluxation	*Improving the scientific understanding of what we do*
Neurophysiological effects/mechanisms of SMT	*will help to improve treatment and identify markers of successful treatment. Will also identify subgroups of patients better responding to SMT. Applications to therapeutics, advancement of evidence based practice*
Evidence of neurophysiological change following manipulation vs sham	*The only way to show that SMT is not just a placebo is to be able to demonstrate demonstrable changes in physiological outcome measures relative to sham procedures*
Investigate the specific mechanisms of action of spinal manipulation on the neuromuscular system.	*Improve treatment efficacy.*
Effect of therapeutic loads on physiological responses in animals and humans.	*Like any other science, we must understand the foundational phenomenon of what we do. Too many clinical studies are leading to ambiguous results because we lack the necessary information from basic science to inform clinical optimization is lacking. An understanding of mechanisms is essential to drive greater clarity in clinical research to inform practice.*
Physiological mechanisms of manipulative therapy -- what happens during a manipulation in terms of inflammatory markers, nervous system changes, etc.	*A better understanding of what manipulation does to the tissues will determine what types of therapy work, why and when, as well as their safety. Focusing on manipulative therapy makes the research relevant to chiropractic in particular (important for chiropractors, chiropractic patients, policy/decision-makers in chiropractic)*
What is the neurobiological basis of SMT?	*Current clinical and epidemiological work essential validates established practices but does not lead to innovation in practice. Innovation requires an understanding of basic physiological mechanisms. Additionally, a scientific rationale is important in encouraging knowledge translation. It is not enough to know that something works. People apparently need or want to know why something works.*
Evidence of altered motor control that improves with chiropractic treatment	*We need to move beyond pain; pain is complex and multi-faceted; current evidence indicates that altered motor control leads to chronic and recurrent neck and back pain; if we can demonstrate that chiropractic care improves motor control, we can provide "proof of principle" to move into clinical studies*
Longitudinal studies of effects of chiropractic treatment on altered motor control	*We need to know how long it takes to "fix" maladaptive motor control strategies to begin to develop evidence based strategies*
somato-visceral response to vertebral manipulations (mechanisms and effects)	*lack of research and difficulty of undertaking clinical research on individuals*
effects of stimulating mechanoreceptors only on the side of a persistent imbalance compared to stimulating on both sides (double blind studies)	

Sub-theme: Biomechanical mechanisms and effects of SMT

Periarticular effects of joint cavitation	*To discover the distal effects of SMT on the proximal, non-articular anatomy/physiology.*
Forces of manipulation	*issues of safety*

THEME: Basic Science Research

Biomechanical mechanisms of manipulative therapy - what happens during a manipulation and what is the effect (at the tissue level)	*Biological modelling allows access and insights to mechanisms not readily observed through experimental research. This has the opportunity to explore mechanism of injury/pain/treatment. A better understanding of what manipulation does to the tissues will determine what types of therapy work, why and when, as well as their safety. Focusing on manipulative therapy makes the research relevant to chiropractic in particular (important for chiropractors, chiropractic patients, policy/decision-makers in chiropractic)*
Controlled human experiments to determine whether spinal manipulative therapy (SMT) is associated with prolonged changes in joint stiffness (or flexibility)	*Need to determine whether SMT has any clinically significant and enduring biomechanical effects*
Defining the influence of systematically varying treatment characteristics (e.g. preload, force impulse amplitude, rate of rise in force and repetition frequency) on biomarkers.	*Load characteristics (e.g. rate and force) are now known to influence outcome.We still do not have a systematic understanding of these factors.*
Is the dynamic coordination between the shoulder and pelvic girdle altered in patients with low back pain and does this change after spinal manipulation?	*Gap in the literature*
The biomechanics of different types of manipulation -- amplitudes and directions of force during different manipulations -- what is the ideal amplitude, rate of force application etc.	*Better knowledge about the forces involved in manipulation will help train future chiropractors and assist existing chiropractors through continuing education*
Manipulation forces used on special populations (i.e., elderly, pediatric, pregnancy)	*Can be used for training purposes and to better understand safety outcomes.*
Impact of variation in speed and force associated with a single type of chiropractic manipulation applied to subjects with low back pain, stratified by age, gender and BMI.	*It is very likely that there are threshold effects that mediate changes when manual therapy is applied to an msk problem. As a result, completing studies to evaluate for potential thresholds is critical to conducting strong future clinical trials. In addition, expertise in manipulation is sought by many manual therapists today. It is important for the chiropractic profession to manage the expertise associated with the complexity of HVLA.*

Sub-theme: Mechanisms and effects of adjunct therapies

Mechanisms of combined approaches	
Investigate the specific mechanisms of action of other treatments (e.g., rehabilitative exercise) on the neuromuscular system.	*Improve treatment efficacy.*
mechanisms/effects of other modalities: soft tissue, etc.	*explain what chiropractors do and how SMT works to patients and stakeholders*
How adjunct therapies work, e.g. like exercise/mobilization/neural mobilization	
Under what mechanism do eccentric exercises work for tendon injuries	*We have recommended eccentric strengthening exercises to patients with tendon injuries for years, I know the literature generally supports but I'm unaware of the mechanism*
Does Active Release Technique applied to the dorsal sacral ligaments alter the patient's straight-leg raise	*The Active Release organization makes this claim at its seminars and in it's instruction manuals, yet there is no data to support this contention.*

Sub-theme: Mechanisms of musculoskeletal disorders

Biomechanics and kinematics in MSK disorders	*Investigate how chiropractic interventions can improve movement and function*
Does joint hypomobility actually exist under natural conditions? What are its mechanisms?	*Chiropractic and other manual therapies are based on the treatment of minor, reversible joint derangement in the form of hypomobility. Yet, this entity has never been verified and characterized in an animal model. All such research to date has assumed the existence and characteristics of this condition.*
Injury mechanism	*Understanding injury mechanism allows you to address patient risk/likelihood of injury/prevention/rehabilitation etc.*
Musculoskeletal pain mechanisms	*We know too little about the mechanisms subserving pain (peripheral and central determinants of pain). More knowledge in this area will help find more appropriate ways to treat it.*
mechanism of joint restriction/fixation	*understanding consequence of joint restrictions on structure and function could contribute to justification of treatment - does restriction matter?*
What impact do static postures have on pain thresholds?	*Sitting and standing are frequently implicated as causes of spinal pain but do they actually have an effect on pain-pressure thresholds*
What is the anatomical/physiological substrate of the subluxation (or whatever one chooses to call the 'manipulable lesion')?	*Practitioners, patients and others want to know what the lesion actually is? Not being able to answer this question in a credible fashion undermines what we now call the 'cultural authority' of the profession.*
Physiological relationships between the musculoskeletal system and the other systems	*Anecdotal evidence shows that some people benefit from chiropractic care for a variety of ailments. We should concentrate our efforts on one organic system (ex: somato-sympathetic reflexes) and study in depth that relationship.*
Can we correlate activity levels with back pain?	*Does general activity and exercise offer a protective effect from spinal pain? And impact of health longer term*

Sub-theme: Physiology of spinal pain

Spine physiology and neurophysiology	*Understanding the normal and abnormal responses of spinal tissues will allow us to better identify dysfunction and properly address it. Investigate the neurophysiological mechanisms of various chiropractic interventions on acute and chronic pain, when intervention is effective at releiving pain.*
determining pain producing structures in back/neck pain	*most back pain is still classified by medical research as idiopathic which leads to low confidence in recommending one treatment approach over another. more research needs to be conducted in order to define the actual structures that are most responsible for producing the pain associated with low back/neck pain so that the rationale for a specific treatment protocol becomes more rational*
The neurophysiology associated to spine disorders(somatic and visceral) and autonomic response	*lack of research in this area specialy related to visceral disorders*
Mechanism of spine pain	*targeted treatment*
Relationship of physiologic and phenomenologic responses to clinical signs, symptoms.	*Mapping physiologic responses to clinically relevant outcomes is important to identifying key features of clinical utility.*
Neuroscience of spinal tissues	*We need to understand how the nervous system receives information from the spine and how it controls and stabilises it. The implications of this work are far reaching, with the potential to transform clinical practice.*
chronic pain mechanisms associated to vertebral mechanoreceptors disorders	*lack of research*
What are the proximate effects of spinal facet joint pain? How do these effects alter the function of the spine?	*Even if hypomobility cannot be proven objectively, spinal pain obviously exists. We need to characterize what local effects spinal pain has on the function of the spine.*

THEME: Clinical Research

Sub-theme: Effects of chiropractic care (general and for specific MSK conditions)

Research Idea	Reason
Efficacy/effectiveness of chiropractic care	*Determine factors (positive or negative) that influence the effectiveness of chiropractic interventions for improving function and relieving pain. Efficacy of treatment protocols; Very few high quality trials in chiropractic. Third party payers may need to base funding according to types of professionals showing better benefit/risk. Determine effective therapies. Much concern exists over the efficacy of chiropractic care. Multicenters RCTs should be conducted to increase knowledge on efficacy. Market share and public trust*
Comparison of mobilization vs manipulation on outcome	*Effectiveness*
SMT+STT vs. SMT only vs. Chiropractic care as a whole (including patient education, etc)	
Prognostic studies	*Determine predictors of outcomes and determinants of clinical outcomes.*
Impact of Chiropractors on overall health status of patients (in addition to MSK)	*If it were possible to design a health promotion program that aimed to improve the overall health of patients (weight reduction, smoking cessation, activity level) and this program was tested within various health professional offices in order to measure compliance and outcomes when the program is delivered by specific professionals, it would likely demonstrate the positive effect that patient education when delivered by a chiropractor can have on the overall health of the general population and move the profession from a therapy based alternative to a health professional with considerable skill in managing the overall health of patients*
What are the MSK and non-MSK outcomes with manipulative therapy	*This may be of interest to chiropractors of all philosophies and may help improve patient care*
Distinguish non-specific effects of manual treatment from any active components of treatment.	*There remains ambiguuity on the potency of manual treatment itself versus nonspecific or 'placebo' effects.*
RCTs with non traditional patient population or hospital populations (patients who are candidate for surgery, rheumatology, etc...	
Study of general health effects of long term chiropractic care on general health	*To determine if chiropractic care has any real general health implications*
Quality of life improvements with chiropractic care	*Health promotion effects of chiropractic care; well being studies ie. depression, mood changes, hormonal effects etc*
Determine the use of "exercise is medicine" and prescribing of physical activitiy by Chiropractors.	*To determine if chiropractors are playing a role in preventing non communicable diseases through Health promotion; specifically PA prescription.*
National longitudinal studies comparing general health as well as condition-specific outcomes of regular versus non-regular users of chiropractic management	*Need to determine whether regular utilization of chiropractic care somehow translates into clinically significantly better health and/or "wellness"*
Can chiropractors contribute to improving environmental awareness and action in their patients?	
Impact of chiropractic care on disability in the general population	
Novel approches to spine function	*We need to lead the way in novel technologies and discoveries surrounding the spine. For example, the potential of basic genetics or physiology experiments is tremendous.*
Effects of chiropractic SMT on spinal pain	*90% of chiros use SMT need to expand evidence on how it works. there are not enough studies using chiropractic manipulation for back pain*
Role of chiropractic care in pre-surgical and post-surgical back pain.	*The original work of Kirkaldy-Willis and Cassidy indicated a significant role for chiropractic in this market. This study was done 30 years ago, yet it appears that chiropractors still have little or no direct access to surgeons who are potentially seeing patients that would benefit from more conservative care. A larger, more up-to-date study in this area is warranted.*
effects of SMT on chronic pain	*identify role of SMT in management of chronic pain*
Well-designed, sham-controlled study of manipulation for any of the typical spinal complaints treated by chiropractors.	
Effect of chiro tx modalities on pain modulation	*Improved understanding of treatment effects and mechanism*
Costovertebral adjustments for rib dysfunction and pain	
effectiveness of chiropractic management of scoliosis	*literature is virtually nil.*
Effect of chiropractic care on ability to perform specific movement patterns	*To perform movements in the safest way requires strength and flexibility; if chiropractic care can be shown to improve the ability to move in a manner that decreases injury risk, it make a very powerful statement*
chiro effectiveness for the treatment of cervical radiculopathies	*Commonly seen in practice*
Chiro treatment of extremity conditions.	*Paucity of evidence assessing manual therapy for conditions other than LBP or NP*
Thoracic treatment	*Paucity of research*
Does specific spinal palpation coupled with specific spinal manipulation produce better results in subjects with a spinal pain complaint (such as neck pain)?	*Traditional chiropractic approaches to palpation and adjustment deserve to be given a chance to properly prove themselves. If they fail to produce substantial difference in clinical outcomes, they should be revisited.*
Compare SMT to SMT and soft tissue therapy and just soft tissue therapy (each with and without exercise therapy as well) for spinal pain	*In practice I certainly don't just adjust patients, I use a combination of therapies. Comparing different combinations to see which produce optimal outcomes is necessary*
Effectiveness of chiropractic care for peripheral musculoskeletal disorders associated to spine (eg: carpal tunnel syndrome,bursitis, tendinitis)	
Impact of chiro care on chronic MSK pain like fibromyalgia	*Commonly treated in practice*
Effect of manipulation on headaches	*It is not studied well enough*
rehabilitation of common extremity conditions e.g., sprained ankles - does chiropractic help reduce recurrence of ankle sprains	*common athletic injury; improve patient outcomes; improve opportunities for chiropractors*
Effects of ultrasound on tennis elbow	*common condition treated in a chiropractors office*
Expanding into new areas - e.g. anterior chest wall issue	
What are the emergency department clinician perceptions and actions regarding non-cardiac chest pain?	*To establish if there is inter-hospital clinician support for treatment of patients with non-cardiac chest pain via on-site conservative care that includes spinal manipulation.*
Stratified care approaches for LBP	*Matched/targeted care may increase the likelihood of better patient outcomes for most common reason for seeing DCs*
long term degenerative process associated to whiplash and the effects of chiropractic care to prevent the degenerative process of the cervical spine	*lack of research on the effects of long term chiropractic care to prevent degenerative process of the spine*

Sub-theme: Effects of chiropractic care (non-MSK conditions)

how does chiropractic help with non-MSK conditions	*This is an area of reported patient outcomes where there is insufficient, if any, supportive evidence; yet it is commonly seen in practice. Can explain to patients and stakeholders, clear up confusion among chiropractors and other stakeholders; positive findings may help provide opportunities for chiropractors*
What is the role of SMT in the management of visceral disorders?	*This is a largely unexplored area which could represent both a strength and 'Achilles heel' for the profession. The profession needs to identify (and reduce) false claims of efficacy, while identifying patients who could derive further benefit from SMT and supportive practices.*
effect of SMT on somatovisceral	*clarification for practioner and patients on possible benefits*
Regulation of autonomic functions	*Determine in which conditions SMT may affect autonomic functions and whether the magnitude of the effects is sufficient to anticipate a clinically significant effect that could be investigated in applied or clinical research projects.*
ADHD outcomes with chiropractic care	*Increasing incidence; notable changes reported by parents/teachers in many children diagnosed but not well quantified; Can supplement and/or potentially offer options other than longterm medication and psychotherapy approaches. Current concerns in medical literature that early medication intervention may have significant limitations in psychological development of personal management skills.*
Quantify lasting effects of SMT on visceral dysfunction	*To discover whether the observable short term visceral functional changes (like BP) have any actual therapeutic value*
Outcome studies of chiropractic adjustments and management of pediatric population with repetitive serous otitis media	*lack of studies to understand the physiological mechanisms to demonstrate results obtained with cervical vertebral manipulations*
Impact of Global (whole body) Health with Chiropractic care	*Support of non-symptomatic care*
Role of chiropractic care on management of chronic msk conditions and assessing impact on general health (non msk)	
What is the effect of chiropractic management of concussion and post-concussion syndromes?	*No known research on this subject*
chiropractic management for patients with concussion and neck pain versus usual medical care	*This continues to be an important public health concern with significant media attention and significant gap in chiropractic research.*
outcome studies of patients suffering from chronic gastritis and stomach ulcers with chiropractic care and management compared to medical care over a period of 6 months	

THEME: Clinical Research

Subtheme: Chiropractic care and older adults

The effect of manipulation for health outcomes and quality of life in the elderly	*Relevant given the aging population and rising health care costs*
Effect of chiro management on degenerative spine conditions (older patient) RCTs	*increasing prevalence and importance*
Compare the use of manipulation with other interventions for spinal degenerative conditions	*Our population is aging, the prevalence of spinal DDD, DJD, and spinal stenosis is only going to increase. We need to have answers for patients as to what will work best for them.*
measures of spinal mobility and quality of life in seniors	*Improved spinal flexibility correlates with aging quality of life.*
spine care in geriatric population to reduce chronic pain	*lack of sufficient research*
long term effects of chiropractic care to prevent degenerative changes in osteoarthritis.	
Immediate pre-post change in pain related to patients with symptomatic osteoarthritis, assigned either to one of two forms of chiropractic manipulation, prognostically stratified by single nucleotide polymorphisms suspected of mediating pain.	*Although controversial, research has suggested that with some forms of musculoskeletal pain, the SCN9A rs6746030 genotype may in part be responsible for differences in individual patients' pain perception. If true, this would clearly affect the outcome of any manual therapy based outcomes trial. We believe that different forms of manipulation are likely to have different outcomes (e.g. Activator vs. Flexion Distraction vs. HVLA) in a given patient, however, this may be mediated by the individual's genetic make-up and should be taken into account.*
Does regular chiropractic treatment lower the incidence of OA/improve OA	*improve patient outcomes; improve utilization of chiropractors*
Studies(RCT's) focusing on manual care for hip (and (knee OA)	*Building on brantinghams work, preliminary work is promising and OA of the weight bearing extremities has significan social and economic burdens*
Become leaders in managing patients with osteoarthritis	*Large burden of disease, aging population, GPs don't do a good job as gate keepers for OA*
Mobility changes in seniors	*seniors able to stay at home longer? decreases in long term care costs*

Sub-theme: Dose-response of SMT

SMT Dose response-relationship on pain, spine stiffness, muscle activity, muscle force, balance, co-ordination etc.	*provide evidence for treatment plans (frequency). advancement of therapeutic protocols; Need more definitive data on constitutes appropriate and/or optimum challenge of therapy for patients with different conditions*
Optimizing treatment dosage (treatment frequency, duration, and procedure force-time profile characteristics) for various clinical disorders.	*Early clinical work shows rate of force development and force amplitude makes a difference. We need to know how to control treatment to achieve optimum clinical results.*
Dose response - for specific diagnoses (ex. degenerative lumbar spinal stenosis - NOT just LBP)	*To inform clinicians, and patients about who may respond to care and over what duration of time.*

Sub-theme: Safety and side effects of chiropractic care

safety of SMT and chiropractic care	*public confidence*
artery tears	*We must continue/support more research with respect to cervical arteries. We should also be present in conferences for neurologists. Neurologists are the most important people to educate regarding the association between chiropractic care and arterial dissections.*
What creates an adverse event?	*We know they are common, but don't know how to avoid them or reduce their frequency or intensity.*
Defining what is an adverse event related to smt	

Sub-theme: Diagnostic testing of spinal conditions

Revisit the theoretical models of spinal affections and seek interprofessional consensus	*There are too many theoretical models of spinal dysfunction and pain; the vocabulary used is also problematic (ex: subluxation). People from different professions must get together and agree to a common basic theoretical model of spinal pain/dysfunction. Common vocabulary must also be adopted.*
Diagnostic value of non-standard examinations such as joint stiffness	*To add evidence-based functional examination processes to the chiropractic evaluation*
Create improved tools for diagnosis and treatment	*Current data shows that our hands are not the best tools for assessing spinal function or for providing consistent care. We risk being left behind by other professions if we do not develop new tools to improve our diagnosis and provision of treatment*
Validate clinical tests against neuromechanical outcome measures	*If we can say that a clinical test strongly correlates with a basic science measure, that test can then be used in clinical studies*
Diagnosis of neck pain	
interexaminer reliability for SMT to determine area to adjust	*consistency of care for patients*
Clinical prediction rule - prognosis and management of neck and low back pain	
Use of diagnostic tests to improve clinical decision making	

Sub-theme: Development of objective outcome measures

Objective outcome measures – gait variability / wearables	
Development of objective clinical outcome measures that can be used to assess treatment outcome.	*Improve treatment efficacy and resource allocation.*
performance based outcomes	*develop objective measures that can assess the response, or lack thereof, to chiropractic management*
How do new measurement techniques (continuous measurements) affect clinical research	

Sub-theme: Patient satisfaction and expectations

Patient expectations on seeing a chiropractor for neck, low back, hip pain etc.	*improve patient-oriented management*
Understanding the impact of patient expectation on recovery	*Evidence points to role of patient expectation on recovery, having a greater understanding will assist in management*
patient satisfaction on seeing a chiropractor for neck, low back, hip pain etc.	*improve patient-oriented management*
patient perspective, experience and expectations on chiro treatments (qualitative research)	*patient centred care- what does the patient have to say*
patient satisfaction outcomes for MD/DC collaborative care (MD/DC inter-clinic referrals)	*support for better integration of chiropractic into mainstream healthcare*
What do patients understand of chiropractic and how does this change how we deliver our message	

Sub-theme: Maintenance/prevention care

Effects of maintenance/wellness care	*positive or negative effects long term. This is common practice for which, as far as I know, there is no real evidence. The profession is in the position of being unable to either defend nor condem the practice because of a lack of evidence.*
Long term chiropractic care (wellness/preventive or maintenance types) and the impact on morbidity or mortality factors - activity levels, etc. Perhaps comparative to chiropractic patients/clients vs. general population.	*Showing long term values of chiropractic in the greater population. Dentists moved the population from only treatment with cavity repair to regular care and prevention. We have benefits that are significant to both the individual and society as a whole over longer term preventative care that we know anecdotally and clinically but little research to document*
Randomized controlled trials (RCTs) of "maintenance" SMT for neck pain	*Need to see if the reported benefit of maintenance SMT on low back pain is applicable to maintenance SMT for neck pain*
Can chiropractic protect against future LBP episodes?	*A well-designed, large population study needs to answer this question which has really never been addressed but is the basis of most of clinical practice.*

Sub-theme: Identification of clinical sub-groups

RCT with subgroups of typical chiropractic patients (LBP, NP, Headache, etc...)	*Improve treatment effectiveness and cost effectiveness Identify responders*
Who responds to chiropractic care?	*We need to develop tools to identify who best responds to our care and find meaningful options for those who do not. This is not the same as understanding the underlying mechanisms*
Understand the mechanisms of who does, and who does not, respond to chiropractic care	*What are the reasons why some people respond to chiro care and others do not? Addressing these questions will help us better understand the mechanisms of SMT as well as of back pain. Basic science to better define responders/non-responders (what care goes with what person at what time), in terms of pain/ disability; e.g. - what is the basic science basis of the start-back tool, Kawchuk responders/non-responders*
Development of objective clinical outcome measures that can be used to identify sub-groups of patients who are more likely to respond to specific treatments.	*Improve treatment efficacy and resource allocation.*
What is non-specific LBP? Should it be divided into sub-groups / conditions?	
Stop all research into "non-specific low back pain"	*This condition does not exist. Clinicians do not see non-specific LBP in practice nor do they treat it. Patients form sub-groups and clusters... investigate each of them.*
How do we identify those exceptional patients with visceral disorders who are likely to benefit from SMT?	*The available evidence suggests that there are patients with visceral disorders who respond very well to SMT, but these people are exceptional. Being able to identify them would bring rationality to practice beyond biomechanical neck and back pain. Well - maybe not, but it would make rationality possible.*
Identify risk behaviours associated with sub-groups of low back pain.	*Occupational, biomechanical, physiological, etc. all play a role in back disorders.*
Social implications if the profession is beginning to be able to define responders/non-responders	

Sub-theme: Chiropractic care for mental health

Impact of chiro care upon mental health	
Impact of developing stronger DC understanding, competency of mental illness, supratentorial aspects of care	
vertebral disorders(back pain and others) and mental illness(depression)	*lack of research in psychosocial area associated with spine disorders*
psychological effects of SMT	*mental state of patients and relevant effects of treatment*

THEME: Health Systems Research

Sub-theme: Integration of chiropractic care into multidisciplinary settings

Research Idea	*Reason*
Benefit of chiro in integrated care	*movement towards team care...role of chiro and benefit and cost effectiveness*
Multidisciplinary integration of chiropractic	*Improve patient access to care and improve chiropractic care effectiveness*
Understanding (interprofessional) integration of the chiropractic profession using a sociology lens	*Need to better understand this bipolar profession to determine strategies to accelerate the cultural change toward EIP.*
Qualitative and economic research looking at multi-disciplinary models to see how integrative care works for spinal pain	*Many chiropractors and policy researchers indicate that multi-disciplinary care is important for spinal pain. However is it cost-effective? In what ways does it or doesn't it work for practitioners and patients?*
health system research that evaluates the use of chiropractors in an enhanced, integrated primary care role	*improve primary care; improve patient outcomes; improve chiropractic utilization; impact decision makers*
How does adding a DC to a health team impact their outcomes?	
Effects of chiropractic integration within mainstream medical programs on resource utilization and patient health status.	*Chiropractic manpower is underutilized primarily because it has little visibility outside of a single treatment modality.*
Assess impact of integrated/collaborative models with chiropractors beyond primary care eg. Impact of advanced chiro surgical – pre & post	
Impact of the integration of chiropractic services in specific occupational populations	
Benefit of integrating chiropractic services into outpatient chronic pain units and interdisciplinary spine centers	*Discover whether the addition of chiropractic care has an efficiency effect on the current orthodox MSK spine care model, exclusive of monetary considerations.*
Hospital based chiropractic care	*What is the impact of having a chiropractor on staff for emergency room wait times. Could chiropractors help screen and provide earlier intervention for patients who are referred for orthopaedic or neurosurgical consultations?*
System Dynamics model to evaluate the role of chiropractic in the Emergency room of a hospital.	*The greatest challenge to designing strong investigations in Health Systems is understanding both the assumptions that are being used when identifying the system and the assumptions about the levers that are actually likely to show change. System Dynamics modeling allows for those assumptions to be explicitly identified and given those assumptions will demonstrate where the levers for greatest change are likely to be. Prior to designing studies that directly investigate these large and complex issues, modeling studies should be used to guide the process.*
Investigate barriers to interdisciplinary collaboration between chiropractors and other health professionals.	*Improve patient management, resource allocation, and health service delivery.*
Chiropractic Care in managing patients in complex care environments	
The outcomes of collaborative care in management of msk conditions	
investigation into Chiropractor/Nurse Practitioner collaborative model of primary care delivery in areas with physician shortages	*in areas where Physicians are not readily available or where demand neccesitates more access to health care professionals the combined skill mix of a chiropractor and a nurse practitioner may offer a unique opportunity to enhance health service delivery in underserviced locations while ensuring quality and safety of service*
Integration in sports setting and work related environment	
evaluate the value of chiropractic in EHC plans	*improve patient outcomes, improve chiropractic utilization; impact decision makers*

Sub-theme: Costs and cost-effectiveness of chiropractic care

Cost effectiveness of chiro inclusion in triage of LBP especially discogenic	*Inclusion*
Cost/benefit ratio	*Data must be analyzed and discussed in order to improve our knowledge of chiropractic Cost/benefit ratio. Important for decision makers.*
Does providing free access to chiropractic reduce costs and morbidiy in the long run	*A definitive study in a Canadian context is needed here.*
Influence of including chiropractic benefits to a population of patients in controlling total health costs.	*Earlier work has implied that system wide effects are observable and beneficial. No comparable Canadian data exists.*
RCTs to assess cost-effectiveness of chiropractic and/or SMT for commonly treated conditions	*Need to assess the cost-effectiveness of chiropractic treatment*
cost efficiency of chiropractic care for acute and chronic neck and low back pain	*evidence for better integration of chiropractic into mainstream healthcare*
cost effectiveness of chiropractic care vs medical care associated to back pain	*lack of recent research on this particular subject*
Cost effectiveness of chiropractic treatment vs. medical care vs. acupuncture vs.... -- over the short and long term	*Will help provide monetary rationale for one type of care over another. That said, looking at the long care cost is key... Will chiro care lead to better quality of life many years down the road and, therefore, lower overall health care costs?*
cost effectiveness of chiro in health teams	
Cost effectiveness of chiro inclusion in triage of neck pain	*Inclusion*
Broader studies of cost effectiveness to include productivity	*Most studies do not include inefficient work behavior due to fatigue and distraction; simple computerized tests could be used to characterize the effect of pain on worker productivity*
Cost analysis of an episode of LBP or Neck Pain	*cost efficiency of Chiropractic*
System Dynamics model of the cost/benefit of chiropractic care and physical therapy when included in the reimbursement structure of the healthcare system for rehabilitation of back and neck pain, compared to when it is not. This includes an analysis of the stage at which chiropractic would be most effectively introduced (prior to entering the medical route or after).	*The greatest challenge to designing strong investigations in Health Systems is understanding both the assumptions that are being used when identifying the system and the assumptions about the levers that are actually likely to show change. System Dynamics modeling allows for those assumptions to be explicitly identified and given those assumptions will demonstrate where the levers for greatest change are likely to be. Prior to designing studies that directly investigate these large and complex issues, modeling studies should be used to guide the process.*
Cost effectiveness of chiropractic services for the management of work related MSK injuries	
RCTs to evaluate the cost-effectiveness of spine care pathways that include the systematic use of chiropractors as either triage clinicians or community-based care providers	*Need to evaluate the benefit to society of integrating chiropractors into the mainstream health care system*
Collaborative care effectiveness / cost effectiveness in general population and WCB and MVA, etc	
Measure impact of policy, policy change, health economics; Example of delisting - what happened before/after policy change	

Sub-theme: Effect of chiropractic care on reducing medical services

chiropractic management and opioid use	*chronic pain and addiction are pressing issues of the day however there is little concrete evidence (to my knowledge) that specifically links Chiropractic management of chronic pain patients with a stastically significant reduction in Opioid use. This research would be very helpful in discussions with governmental organizations with regards to managing chronic pain patients especially when addiction issues are present*
Decreased pain medication use during critical time periods (i.e., pregnancy, post-postpartum, pediatrics)	*Pain medication use is a growing concern, especially in these vulnerable populations. If chiropractic care is found to help with conditions that these medications are prescribed for, then this can help alleviate some of the problem.*
Does the addition of chiropractic services to a publicly funded system: i) decrease surgical waitlists, ii) reduce emergency department wait times?	*explore the role of chiropractic in publicly funded systems for potential integration*
Chiropractic patients and their utilization rate of medical health care	*reduction in drug costs and hospital, well being*
System-wide effects of chiropractic care (wait times, health care costs, etc)	

Sub-theme: Chiropractic care vs. other care

Chiropractic Care versus traditional Medicine in the management of NMSK disorders	
medical care vs chiropractic care for low back pain	*medical treatment is funded by government, chiropractic is not, need studies comparing the two to show which is more cost effective*
Large-scale surveillance studies to compare the costs, risks (adverse events), and benefits (use/avoidance of other health services) of chiropractic care versus medical care for common musculoskeletal conditions	*Need to determine if increased utilization of chiropractors has a benefit of averting iatrogenesis/adverse events from pharmacotherapy and/or dollar costs of expensive medical diagnostic and therapeutic interventions*
efficiency (time/cost) for chiropractic vs typical MD care (neck, back, joint pain) etc.	*evidence for better integration of chiropractic into mainstream healthcare*
Pragmatic trials	*Very few high quality phase 4 trials in chiropractic. Third party payers may need to base funding according to cost/benefit evaluation*
are patients who receive chiropractic care vs medical care more active and as a result have a lower risk of osteoarthritis or heart attack or stroke	*a small investment in chiropractic care might same millions in less cost to treat arthritis, heart attack or stroke*

Sub-theme: Access and barriers to chiropractic care

Understanding access and barriers to patients seeking chiropractic care	*utilization of chiro services has remained relatively flat, why? Shape the direction of the profession and change attitudes within the profession. Determine if there are gaps in care for different populations and highlight the needs if there are under serviced groups.*
Access to chiropractic care: work injuries	*We should have more national data on access to chiropractic care for injured workers. This should include data comparing the efficacy of chiropractic with other types of care.*

THEME: Health Systems Research

Access to chiropractic care: MVAs	*We should have more national data on access to chiropractic care for injured people after a MVA. This should include data comparing the efficacy of chiropractic with other types of care.*
Urban vs. rural – access to care, models of care	
An agent-based model to evaluate the different decision-making strategies of individual patients based on economic choices for their musculoskeletal care, and the resultant costs/benefits.	*Agent-based modeling allows models to be developed of relative populations of patients with different economic incentives, based on different economic circumstances and health care needs. This results in a dynamic population moving through a system. The intent is to guide policy makers to understanding the optimal ratios and likely economic and health outcomes for decisions related to access to subsidized healthcare by patients.*
What do other clinicians/professions understand of chiropractic and how does this change how we deliver our message	
Credibility of chiropractors: determine strategies to impact credibility; implement and assess outcomes of change	*From a cultural point of view, chiropractors lack credibility compared to MDs. The profession should study this more carefully and develop ways to increase this credibility (ex: would obaining prescribing rights increase chiropractors' credibility?)*
Look at other jurisdictions that may provide data to understand increase access, quality to groups (military, injured workers, 3rd party)	
Patient choice in health care provider	*even though the publicly funded system provides access to many therapies, people still choose to see chiropractors using their own money, workers compensation or benefits packages. Why do patients value care? How can we make care more accessible?*
Attitudes of other professions towards the chiropractic profession	*what do we need to do to earn the respect of our partners in health care delivery*

Sub-theme: Practice patterns, practice behaviours, and guideline implementation

Evaluating impact of guideline implementation in clinical practice	*Need to show guidelines impact on care and patient health outcomes*
Impact of guidelines on patients - Natural experiment to compare those following guidelines already vs those who do not	
translate guidelines into practical treatment strategies	*current guidelines for management not useful*
assessing the quality of chiro care in practice	*limited information*
Knowledge translation research -- what research is used, why and when	*Improved used of knowledge stands to improve patient care, policy-related decisions, and patient outcomes*
Do chiropractors differ in their ability to adopt a EBM perspective and how can we best help them do so.	
Understanding factors within the profession and within professionals to accept change	
How can raise the confidence of our colleagues to look at new ideas, embrace change	
understanding patient profiles in chiro offices	*limited knowledge of profiles and comparisons across jurisdiction*
Patient care trends	
imaging and chiropractic	*define role and need and funding e.g. MRI*
Patient-centeredness of chiropractic care	*Conduct studies that ask patients about how patient-centered their experience with their chiropractor. Patient centeredness is a key component to quality of health care.*
Looking "inside the box" - look inside the profession - to understand where we need go	
Profession self reflection: identifying "practice types" and i) assessing their specific outcomes, ii) if difference, then why/what explains it, iii) enhancing quality and safety of care	
Determine cultural identity of chiropractic profession.	*Profession is divided and nothing will move forward until it finds its identity.*
What is the language of chiropractic? Language is perhaps the highest expression of culture and more than any other expression distinguishes one cultural group from another. How does our language set us apart (and perhaps keep us apart) from others?	*An area of research which is largely neglected but which helps us to understand ourselves in ways which can be articulated.*
Where chiropractic sits in terms of conventional vs. unconventional care	*Has social and cultural implications -- if seen as an alternative profession then its members may not be keen on furthering research so as legitimize chiropractic becoming main stream. Knowing this would help focus knowledge translation efforts*
Measurement of disability/functioning in clinical practice	
How to better provide education to patients - to be better informers to the patients	

Sub-theme: Third-party payers

Comparison studies on government and/or private insurance coverage on chiropractic care in different provinces/territories/states.	
Models of care integrating/coordinating public and private payers	*The ideology of the single payer public system generally inhibits this. Health care system finances are such that it seem inevitable that the public system will need to take advantage of the resources in the private system.*
impact of funding on chiropractic	*access inequity*
Comparison studies on types of chiropractic care (i.e. spinal adjustments, orthotics, compression hosiery, acupuncture, diagnostic imaging, etc) that are covered through government and/or private insurance in different provinces/territories/states.	
Mixed payment models (private/public/co-pay models)	
Develop models that would integrate private-public pay within context of health system	
Effect of payment structure (e.g. capitation) on patient health, chiropractic utilization etc	
Etiology of neck pain in compensation systems	
RCTs of different but commonly administered forms of "chiropractic treatment" on work absenteeism, return to work, and other work-related functional outcomes	*Need to look at the efficacy of chiropractic treatment including SMT in terms of clinically important functional outcomes and not just pain reduction*
How is research cherry-picked by government/insurance to the profession'd benefit/detriment…	
SMT and productivity in workforce	*cost to insurance and industry*
MSK work related injuries	*this is our core practice*
spine care associated to prevention of back pain in industry	*prevention is the key to reduce absenteeism in industry*

Sub-theme: Chiropractic education/training

SMT learning best practice	*we need tools to quantify best learning*
Does clinician tactile perception influence the kinetics and kinematics of SMT?	*Gap in the literature*
Impact of implementing best practice and guidelines in chiropractic schools	*Might lead to larger and more sustainable change among future care providers*
Do special designations improve care?	

Sub-theme: Models of chiropractic care

Models of care that include chiro	*need to demonstrate value to health care system*
Compare guideline based treatments for spinal pain with usual care provided by either a GP, PT, or DC (RCT)	*There is great emphasis on the use of guidelines, does following them actually produce better outcomes when compared with usual care*
Patient outcomes with various models of care -- do patients get better (pain + overall well being), within what time frame and frequency of care with different models of care (pain-based care, maintenance care, low vs. high frequency care)	*This are is of interest to all types of chiropractors, regardless of philosophy. This will help draw in chiropractors who are not necessarily pain-based (help get them more interested in research?? -- ultimately getting them more interested in research will help improve patient care)*
High volume vs. low volume practitioner effectiveness	
Investigate the efficacy and patient preferences regarding different models of care (solo practitioner, chiro among a group of allopathic practitioners, chiro among a group of CAM practitioners)	*This will help inform the ideal model for care and justify whether the system should fund chiro care as part of care teams*
Triage versus non-triaged care on MSK conditions	
Expanding patient services and relative value of chiropractic by incorporating public health initiatives/education into practice.	*Chiropractic manpower is underutilized primarily because it has little visibility outside of a single treatment modality.*
Role of chiropractic as care managers coordinating health systems resources.	*Chiropractic manpower is underutilized primarily because it has little visibility outside of a single treatment modality.*
Work disability prevention as a career track for chiropractors.	*Chiropractic manpower is underutilized primarily because it has little visibility outside of a single treatment modality.*
Use of advanced practice chiropractors in managing complex spinal disorders	

THEME: Population Health Research

Sub-theme: MSK population health

Research Idea	Reason
Importance of back pain to Canadian society: Impact studies, Direct / indirect costs, Disability, Lost productivity, Opioid dependence	
Quantifying the impact of MSK health on the ability to self manage diabetes (and other chronic conditions)	*As practitioiners continue to be organized into teams, our ability to contribute to the health care systems biggest challenges should be explored. We assert that improved MSK health can improve a diabetic patients ability to walk, exercise, etc.*
Relationships between obesity / nutrition / physical activity / other factors	*This is an area of major concern. An expertise in this area would allow chiropractors to be involved in multidisciplinary teams.*
managing neck pain at a population level	*limited but quality evidence suggests population based public health impacts outcome*
A Framingham-type study to be conducted for musculoskeletal pain.	*Musculoskeletal pain is extremely complex, spanning issues such as psychological make-up, support systems, cultural diversity, environmental factors such as occupation etc. This level of complexity can only be looked at through very, very large data bases over an extremely length period of time. As a result, a Framingham-type approach should be considered for a 40 year longitudinal study that includes everything from genetic to physical testing to MRI imaging to participants on a regular basis.*
What is the prevalence of spinal pain in different subgroups in Canada (seniors, children, lower income, First Nations, Canadian military and veterans)	*So often we depend on data from other sources, it would be nice to see chiropractic researchers leading the way on this type of research as it impacts Canadians substantially and provides a large proportion of the patients that chiropractors see*
Identify at risk populations	
Research in recreational activities	*Prevention of injuries*
Effect of wait time on MSK conditions	
Do the Gallup equivalent from the USA here in Canada.	
identification of high risk population for disability	*to develop prevention strategies*
Biopsychosocial impacts and outcomes of msk pain, impact of mental health issues	

Sub-theme: Management of co-morbidities

Self-management strategies to address MSK co-morbidities	*No MSK profession has really taken a lead*
Effect of co-morbidities on recovery rate, preferred method of treatment	
How does managing chronic disease fit in with chronic LBP, acute LBP etc	

Sub-theme: Determinants of health

How does lifestyle influence clinical outcomes?	
What is the effect of culture on the experience of neck pain?	
Understanding MSK disorders social determinants	*Poorly investigated area. However, good evidence that Social Determinants of Health are important over the life course for several health disorders*
Looking at social determinants of health in relation to MSK (cultural, education, poverty etc); How might these determinants effects who we treat and when and how	
Risk studies to investigate determinants of health	

Sub-theme: Chiropractic care for special populations

Look at different clinical populations (military, First Nations, veterans, geriatric, RCMP, disadvantaged, pediatrics)	
How can we improve the access to chiropractic care for marginalized patient populations?	*Those with lower or no income have limited access to chiropractic care due to limited funds, and yet they will still often have msk pain. We need to develop models to help these patients.*
Which geographical areas of Canada or the larger world are under- or over-serviced by chiropractors?	*There is currently no resource for practitioners or health care planners which allows identification of under- and over-serviced areas. This leads to inefficient distribution of resources and is harmful to patients and practitioners.*
Access to care for MSK complaints in aboriginal populations.	
Improvement in quality of life of aboriginal people by the inclusion of chiropractic care in their local health facilities.	
Access to chiropractic care: special populations	*We should identify one special population (ex:older people) and study what limit their access to chiropractic care.*
Dc utilization by special populations (urban, rural, proximity to clinic, school)	
Chiropractic care in the overall well being of certain populations (like pediatrics??) (measures beyond pain)	*This may show that chiropractic care has no effect; however, given that many chiropractors treat a lot of pediatric patients, this will help inform whether these treatments are substantiated*
Creation of a national registry of chiropractic patients	*Only through the accumulation of very large sample sizes can we meaningful search for variables to better identify characteristics of responsive subgroups for any/all of the conditions we currently treat, as well as all/any of the interventions that we currently administer.*

THEME: Population Health Research

Patient priorities

What are the priorities of patients?	*Researching the chiropractic priorities is ill advised. History has taught us that - there are over 200 techniques systems which have followed various "Chiropractic Priorities." Rather, it is the patients chiropractors treat or should be caring for that ought to be the focus of a research direction. This group needs to think well beyond the limited question they have posed to the benefit of the "Chiropractic Profession" and ask the question "Who, Where, What, How, When" in relation to the patients or future patients with back disorders the profession hopes to care for. If chiropractors are seen as the non-surgical spine care experts in all of the health care system the question posed here becomes irrelevant.*
